# The effect of repetitive flexion and extension fatigue loading on the young porcine lumbar spine, a feasibility study of MRI and histological analyses

**DOI:** 10.1186/s40634-017-0091-7

**Published:** 2017-05-12

**Authors:** Olof Thoreson, Lars Ekström, Hans-Arne Hansson, Carl Todd, Wisam Witwit, Anna Swärd Aminoff, Pall Jonasson, Adad Baranto

**Affiliations:** 1 Department of Orthopaedics, Institute of Clinical Sciences at Sahlgrenska Academy, University of Gothenburg, Gothenburg, Sweden; 2 Department of Radiology, Institute of Clinical Sciences at Sahlgrenska Academy, University of Gothenburg, Gothenburg, Sweden; 3 Institute of Biomedicine at Sahlgrenska Academy, University of Gothenburg, Gothenburg, Sweden; 4 The Carl Todd Clinic, 5 Pickwick Park, Park Lane, Corsham, SN13 0HN UK; 5 Orkuhúsið Orthopedic Clinic, Reykjavik, Iceland; 6 Department of Orthopaedics, Sahlgrenska University Hospital/Sahlgrenska, SE-413 45 Gothenburg, Sweden

**Keywords:** Fatigue, Repetitive loading, Animal experimentation, Spine, MRI, Intervertebral disc, In vitro, End plate histology, Growth zone injury

## Abstract

**Background:**

The biomechanical mechanisms of failure of FSUs have been studied but the correlation of repetitive flexion and extension loadings to the initial phase of fatigue in young FSUs are still not known. The purpose of the study was to examine the fatigue results of low magnitude repetitive flexion and extension loading on porcine lumbar Functional Spinal Units (FSUs) with Magnetic Resonance Imaging (MRI) and histology.

**Methods:**

Eight FSUs were subject to repetitive pivot flexion and eight to extension loading by a protocol of 20 000 cycles at 1 Hz with a load of 700 N. All loaded FSUs (*N* = 16) were examined with MRI and histology post loading. Three FSUs were examined with MRI as controls. Further three FSUs were non loaded histology controls.

**Results:**

Fifteen (94%) of the loaded FSUs have decreased MRI signal in the growth zone of the superior vertebra and 12 (75%) in the inferior vertebrae. Fourteen (88%) FSUs have increased signal in the superior vertebral body. Fourteen (88%) FSUs have a reduced signal in all or any endplate. The histology morphometry displayed that the unstained parts of the epiphyseal growth zone were larger among the loaded FSUs (mean 29% vs 4%) and that the chondrocytes in the endplate and growth zones had abnormal structure and deformed extracellular matrix.

**Conclusion:**

Repetitive loading of young porcine FSUs in both extension and flexion causes concurrent MRI and histological changes in the growth zones and endplates, which could be a first sign of fatigue and an explanation for the disc, apophyseal and growth zone injuries seen among adolescent athletes.

## Background

Clinical studies have reported higher levels of low back pain (LBP) and spinal abnormalities among athletes compared to controls (Sward et al. 1991; Kujala et al. 1996; Baranto et al. 2006; Bergstrom et al. 2004; Hangai et al. 2010). This is believed to be due to different demands and loads to the spine in sports compared to normal daily activity. The spine, during sporting activities of both contact and non-contact kind, is subjected to many complex loading patterns. These patterns could potentially be harmful, especially when fatigue and overuse limits are reached. Repetitive loading in axial, flexion, extension and rotation have all been linked clinically to the development of both spinal injuries and LBP (Balague et al. 2012). It is known that many athletes develop spinal abnormalities already at an early age (Baranto et al. 2006; Hellstrom et al. 1990; Sward et al. 1990a; Sward et al. 1990b; Granhed & Morelli 1988) and that after the growth spurt the frequency of radiological changes reported have been shown to increase (Jackson et al. 1976; Goldstein et al. 1991; Tertti et al. 1990), which implies that the spine is especially vulnerable during adolescence (13-18 years of age).

Repetitive axial spinal loading affects the disc height (Gooyers et al. 2012; Dimitriadis et al. 2011) and the fluid levels, in both experimental models as well as in clinical studies (Cheung et al. 2003; Masuoka et al. 2007). There is also strong evidence that repetitive loading affect both discs and vertebrae, and can cause pathologies such as disc degeneration and disc hernias depending on load attributes (Parkinson & Callaghan 2009; Tsai et al. 1998; Yu et al. 2003). Baranto et al. (Baranto et al. 2005a) showed that Functional Spinal Units (FSUs) from young pigs were most vulnerable in the growth zone when they were loaded in flexion or extension to failure. Other loading patterns have also resulted in injuries in the endplates and growth zones in both young porcine spine and human cadaveric studies (Baranto et al. 2005a; Baranto et al. 2005b; Thoreson et al. 2010; Qasim et al. 2012). Loading in flexion of the spine is linked to the development of disc hernia (Wade et al. 2014) possibly through endplate failure (Wade et al. 2015). Strong evidence indicates that a combination of repetitive flexion and extension increase the risk of injury of the disc (Balkovec & McGill 2012; Callaghan & McGill 2001). This implies that the growth zone and the endplate of the disc are extra vulnerable for both initial fatigue and concluding failure due to overuse loading in the young spine.

To understand the mechanical loading conditions one must consider loading duration, frequency, magnitude and motion, all contribute in their specific way to both fatigue and failure stress of the FSU. Each FSU also has inherent properties contributing to its stiffness and strain capacity such as bone mineral density (Huber et al. 2010) and geometry. Furthermore the age of the experimental subject is also an important factor. The spine of young pigs sustain only 25% of the load that require for injury compared to the adult spine (Karlsson et al. 1998).

There is a great heterogeneity regarding study protocols in the literature and no established gold standard to achieve best clinical implication. Considering six cadaveric studies with axial repeated loading, the protocols ranged between 0.25 and 5 Hz in frequency, 1,000–1,290,000 cycles in duration and 404–7,100 N in peak load magnitude (Huber et al. 2010; Hardy et al. 1958; Liu et al. 1983; Brinckmann et al. 1988; Gallagher et al. 2005; Hansson et al. 1987). Repetitive flexion studies are few but Callaghan et al. (Callaghan & McGill 2001) had a test protocol regarding disc herniation at a frequency of 1 Hz, 34,974–84,220 cycle duration and 260–1472 N in peak load magnitude (Callaghan & McGill 2001).

There are, to our knowledge, no experimental reports published that have studied the fatigue effect of repetitive flexion and extension loading of young lumbar spines. This lack of knowledge limits the understanding regarding possible causes of the development of spinal abnormalities during adolescent sporting activities.

The aim of the present study was therefore to determine the effect of repetitive loading in flexion and extension to the disc and vertebrae of young porcine. Endpoints of the study were: 1. MRTI T2 signal differences between loaded and unloaded FSUs. 2. Morphometric analyzation of unstained area in the endplate and the growth zone of loaded and unloaded FSUs. 3. Location comparison between MRI and histological examination results. The hypothesis was that repetitive loading will give rise to changes in the disc and growth zones that will be visible in both MRI and histological analyses.

## Methods

Seven young, healthy, male domestic porcine with an age of 6 months and weight between 75 and 80 kg were used in the present experimental study. The porcine lumbar spines were acquired through a local abattoir (Dalsjöfors Kött AB, Sweden) where all pigs had undergone standard health inspection. The muscles were removed from the lumbar spines, while the posterior bony elements, capsular structures and ligaments were left intact (Baranto et al. 2005a; Baranto et al. 2005b). Nineteen FSUs, seven at the L2–L3 level, seven at the L4–L5 level and five at the Th12–L1 level, were collected. In order to facilitate mounting of the FSUs, the intervertebral discs from the cranial vertebra and from the inferior endplate of the caudal vertebra were removed. The superior part of the cranial vertebra and the inferior part of the caudal vertebra were then mounted in testing cups and stabilized with polyester putty. The vertebras were mounted to achieve parallel surfaces that were perpendicular to the compression axis according to protocol by Baranto et al. (Baranto et al. 2005a). The FSUs were placed in plastic bags to minimize dehydration and were stored at +8 °C in a refrigerator between preparation and testing. The FSUs were divided into two groups with eight FSUs in each and three histological controls.

Three FSUs were examined pre-loading and used as controls to all MRI examinations. Three un-loaded FSUs from level Th12–L1 and L4–L5 from separate pigs were used as controls to the histological examination.

**Fig. 1:**
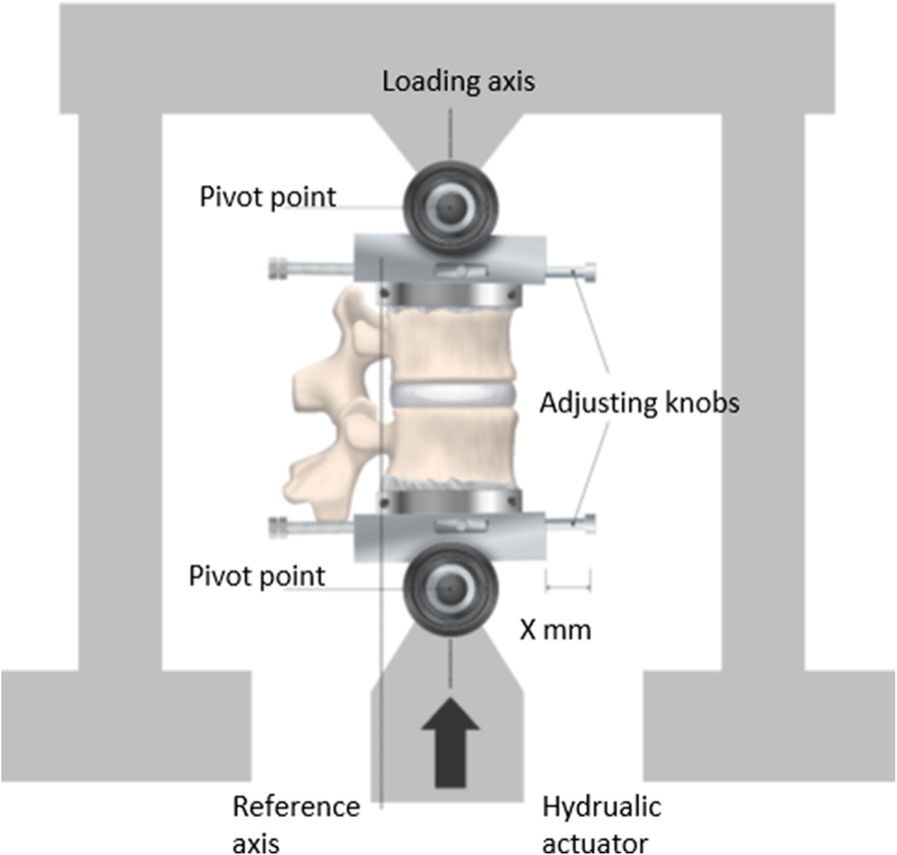
Schematic view of the experimental set up in the MTS testing machine

The biomechanical test is a modified version of the procedure previously used by Lundin et al., Baranto et al. and Thoreson et al. (Baranto et al. 2005a; Thoreson et al. 2010; Lundin et al. 2000). Each FSU was mounted in a servo-hydraulic universal testing machine (MTS Test Star, Minneapolis, MN, USA). The testing cups were free to move around the pivot points during the test. Eight FSUs underwent the repetitive flexion test and eight FSUs were used for the extension test (Fig. 1).

**Fig. 2:**
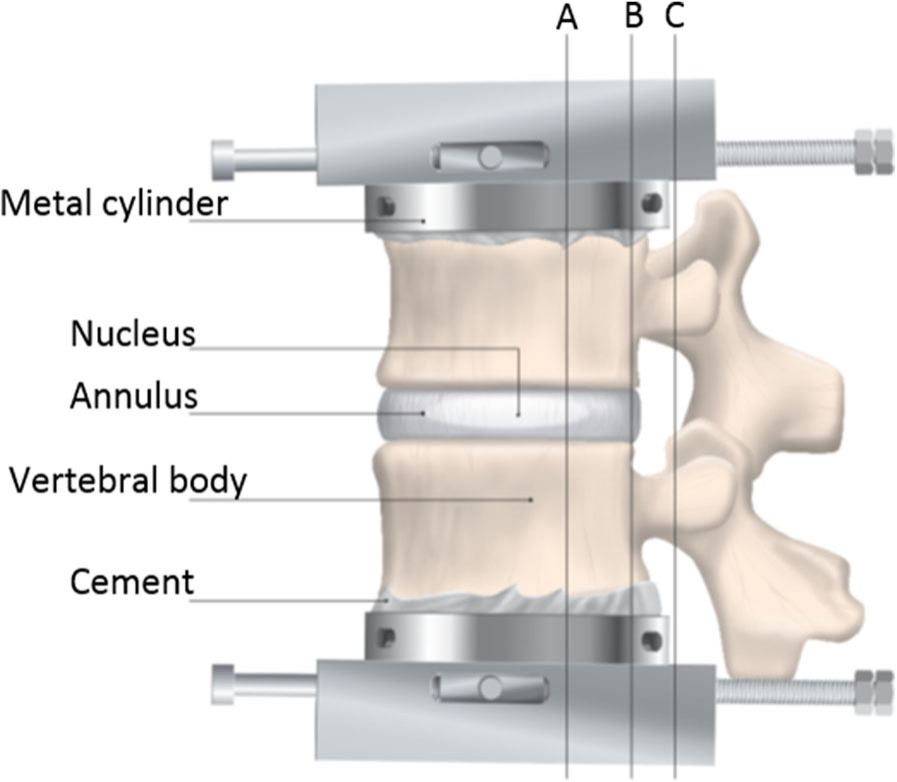
Sagittal location of pivot point according to flexion (**a**) and extension (**c**) in relation to the most dorsal point of vertebrae (**b**)

A procedure according to Baranto et al. (Baranto et al. 2005a) was used to achieve adequate flexion and extension angles (Fig. 2). In the flexion test the FSUs were mounted with the load center anterior from the most dorsal part of the vertebra to allow flexion with a mean angle of 12 (range 10–15) degrees. In the extension test the FSUs were mounted with the load center located posterior from the most dorsal part of the vertebra to achieve adequate extension, which occurred with a mean angle of 9 (range 8–10) degrees. During the tests the FSUs were wrapped in saline-soaked gauze, to prevent dehydration of the discs, and the gauze was continuously hydrated.

The applied force was set to 700 N which is 30–35% of FSU maximum loading capacity before injury in the specific loading mode, i.e. flexion and extension, according to earlier studies (Baranto et al. 2005a; Thoreson et al. 2010). The FSUs were loaded in axial sinusoidal cyclic compression at a frequency of 1 Hz with a duration of 20,000 cycles, which took approximately 5.5 h. The duration was chosen due to its correlation to the total distances covered in games of Australian football (Burgess et al. 2006) and soccer (Barros et al. 2007). The frequency was chosen to its correlation to the stride frequency during a normal distance run (Cavanagh & Kram 1989).

All FSUs were examined with a 3.0 Tesla MRI (Intera, Philips Medical Systems, Netherlands) in Gothenburg. The loaded FSUs were examined post-loading and the controls without any loading. The segments were examined within an extremity coil at a field strength of 3.0 Tesla. Study protocol was sagittal and transversal T2 images (SPAIR Sagittal at a frequency of 229.6 Hz; TR 2537; TE 35), with a field of view of FH: 170 mm, AP: 170 mm, RL: 89 mm. A matrix of 512 × 384 and a slice thickness of 3 mm with a 0.5 mm gap were used in all sequences. The MRI radiographs were examined in a blinded manner by two of the authors independently in a test re-test manner. When assessment was not agreed a second retest was done side by side to reach agreement.

After the MRI examinations, the FSUs were frozen and stored in a -20 °C freezer for at least 12 h. The frozen specimens were then sectioned into 3–4 mm thick sagittal slices, using a bench saw. Each slice was macroscopically checked for iatrogenic damage. The histological preparation were done at registered laboratory (ISO/IEC 17025:2005 and EA2/15). The slices were decalcified (EDTA), dehydrated (ethanol and isopropanol), fixed in paraffin (wax medite pure paraffin), and cut in 4 μm thick sections using a microtome. The samples were stained with Mayer’s Haematoxylin-eosin and Alcian blue- van Gieson solution (Baranto et al. 2005a; Baranto et al. 2005b). Eight histological sections from each FSU were examined for injuries, at a magnification x 1- x 40 by a specialist in histology in a blinded manner with a Leitz DMR microscope. Morphometric analysis of the area per cent of absent staining of the epiphyseal cartilage tissue, comprising the resting, proliferative hypertrophic zones, was done with a Leica QWin Pro system (Leica Biosystems GmbH, Wetzlar, Germany).

The injuries and fractures were defined according to previously protocol by Baranto et al. (Baranto et al. 2005a; Baranto et al. 2005b). Fracture of the endplate was defined as a fracture line through the endplate itself. A fracture in the growth zone was defined as a separation or widening of the growth zone with separation of the endplate from the vertebral body. Signal intensity was evaluated in the growth zone, vertebral body, end plate and disc in each FSU. The signal was evaluated as decreased, normal or increased according to location and as compared to controls. Disc height was determined as normal or reduced. Disc degeneration was graded according to Pfirrman et al. (Pfirrmann et al. 2001) which is a five level MRI grading system for the assessment of lumbar disc degeneration.

### Statistics

Data were statistically described in terms of mean and standard deviation (SD), median and range, or frequencies and percentage when appropriate. The study was conducted as a feasibility study and was thereby low in sample size.

### Data availability

Availability of data and materials can be acquired through contact with the corresponding author.

## Results

The basic characteristics are shown in Table 1.

**Table 1:** Basic characteristics of the FSUs

FSU	Load	Angle	Level	Distance from B-line
1	Flexion	10	L4–L5	10
2	Flexion	12	L2–L3	11
3	Flexion	12	L4–L5	12
4	Flexion	10	L2–L3	12
5	Flexion	10	L4–L5	13
6	Flexion	13	L2–L3	12
7	Flexion	15	L4–L5	12
8	Flexion	15	L2–L3	14
9	Extension	9	Th12–L1	10
10	Extension	10	L2–L3	10
11	Extension	10	L4–L5	10
12	Extension	9	Th12–L1	10
13	Extension	9	L2–L3	10
14	Extension	8	Th12–L1	10
15	Extension	9	Th12–L1	10
16	Extension	10	L4–L5	10

Angle in degrees. *FSU* Functional spinal unit. Distance in mm. B-line as in Fig. 2

**Fig. 3:**
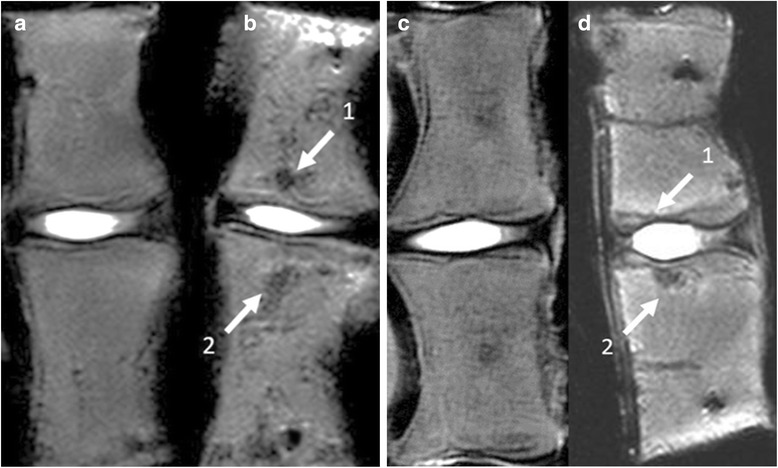
Un-loaded control (**a**) and FSU after repetitive flexion (**b**). Decreased signal in both the superior (1) and inferior growth zones (2) and endplates in the flexed FSU. Un-loaded control (**c**) and FSU after repetitive extension (**d**). Reduced signal in the superior growth zone (1) and in inferior end plate (2) in the extended FSU

All control FSUs display normal MRI signal in both the disc and the vertebras. The MRI evaluation is shown in Table 2. The MRI findings are seen in Fig. 3. Fifteen out of sixteen (94%) loaded FSUs have decreased signal in the growth zone of the superior vertebra. Fourteen (88%) FSUs have increased signal in the superior vertebral body. Twelve (75%) FSUs have decreased signal in the growth zone of the inferior vertebra. Eight (50%) FSUs have increased signal, meanwhile three have decreased signal and one FSU have both decreased and increased signal dependent on location in the inferior vertebral body. No fractures were visible on the MRI images.

**Table 2:** MRI signal of the vertebral bodies of the loaded FSUs

FSU	Superior growth zone, V/D	Superior vertebral body, V/D	Inferior growth zone, V/D	Inferior vertebral body, V/D
1	-/-	+/0	0/0	0/-
2	-/-	+/0	-/-	-/0
3	-/-	0/0	-/-	0/0
4	-/-	+/+	-/-	+/+
5	-/-	+/+	-/-	0/0
6	-/-	+/+	0/0	0/0
7	-/-	+/-	-/-	0/0
8	-/-	+/+	-/-	+/+
9	-/-	+/+	0/0	0/+
10	-/0	+/+	-/-	+/-
11	-/-	+/0	-/-	+/+
12	0/0	+/+	0/0	+/+
13	-/-	+/+	-/-	+/0
14	-/-	+/+	-/-	+/+
15	-/-	0/0	-/-	0/+
16	-/-	+/+	-/-	-/0
Control 1	0/0	0/0	0/0	0/0
Control 2	0/0	0/0	0/0	0/0
Control 3	0/0	0/0	0/0	0/0

According to superior or inferior vertebra and ventral (V) or dorsal (D) location of the FSUs. Grading as reduced (-), normal (0) or increased (+)

The MRI disc results are presented in Table 3. Disc height reduction was seen in 11 FSUs (69%). Reduced signal in both the cranial and caudal endplates is seen in seven out of eight (88%) flexed FSUs. In the extension group no effect was visible in the superior endplate while six out of eight (75%) FSUs had a reduced endplate signal in the inferior endplate. In total fourteen (88%) FSUs have a reduced signal in all or any endplate. Thirteen discs have a Pfirrman grade 1, which is a normal disc, and three discs have a grade 2.

**Table 3:** MRI of the discs of the loaded FSUs

FSU	Pfirrman grade	Disc height	End plate signal S/I
1	1	-	-/-
2	2	0	-/-
3	1	-	-/-
4	1	-	-/-
5	2	-	-/-
6	1	-	-/-
7	1	0	-/-
8	1	-	0/-
9	1	-	0/0
10	1	0	0/-
11	1	0	0/0
12	1	0	0/-
13	1	-	0/-
14	2	-	0/-
15	1	-	0/-
16	1	-	0/-
Control 1	1	0	0/0
Control 2	1	0	0/0
Control 3	1	0	0/0

According to superior (S) or inferior (I) vertebra of the FSUs. Grading as reduced (-), normal (0) or increased (+)

**Fig. 4:**
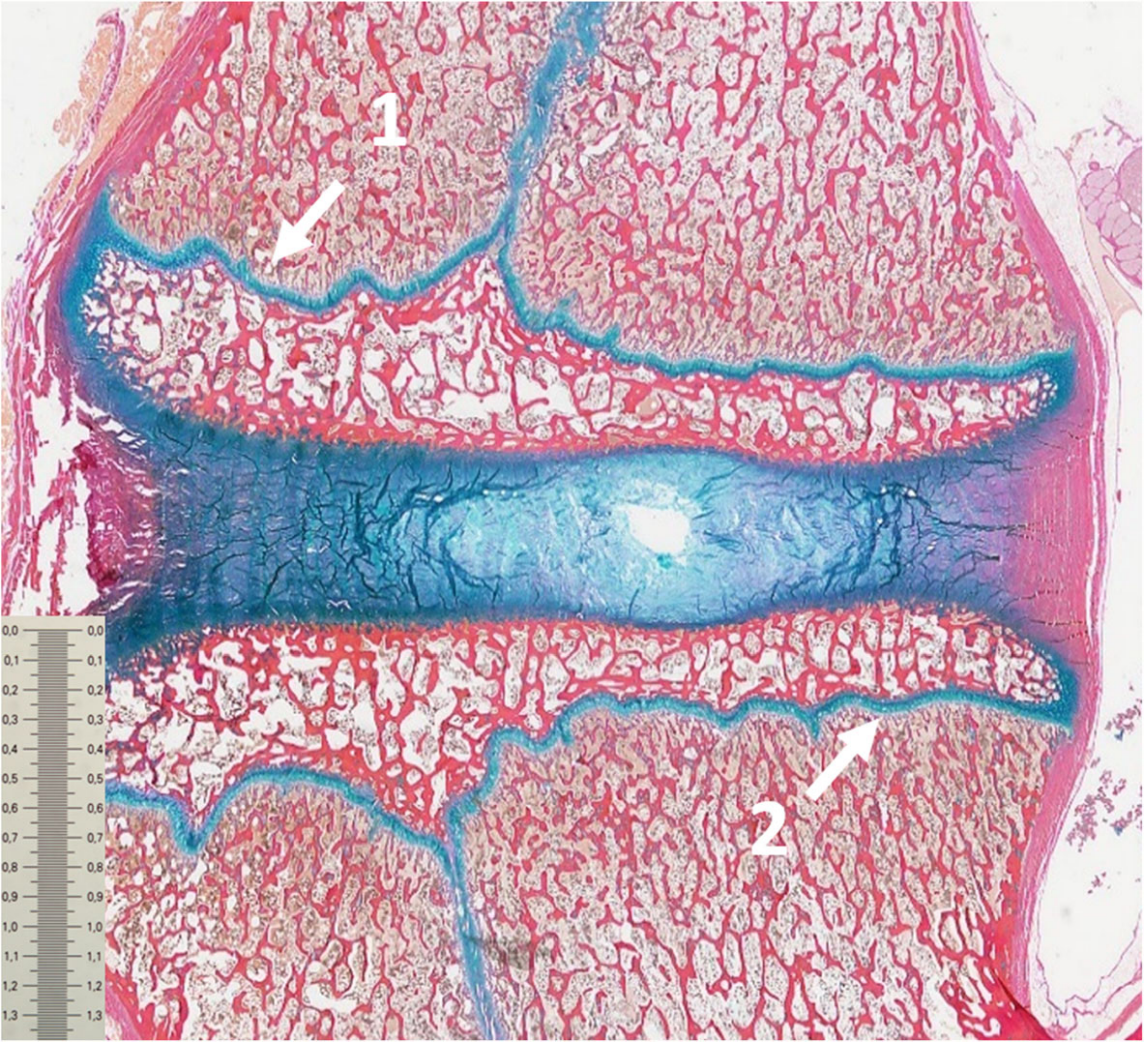
Histological overview of a flexed FSU. Highlighted are the cranial-anterior growth zone (1) and the caudal-posterior growth zone (2). Sizebar in cm. Haematoxylin-eosin and Alcian blue solution stain

**Fig. 5:**
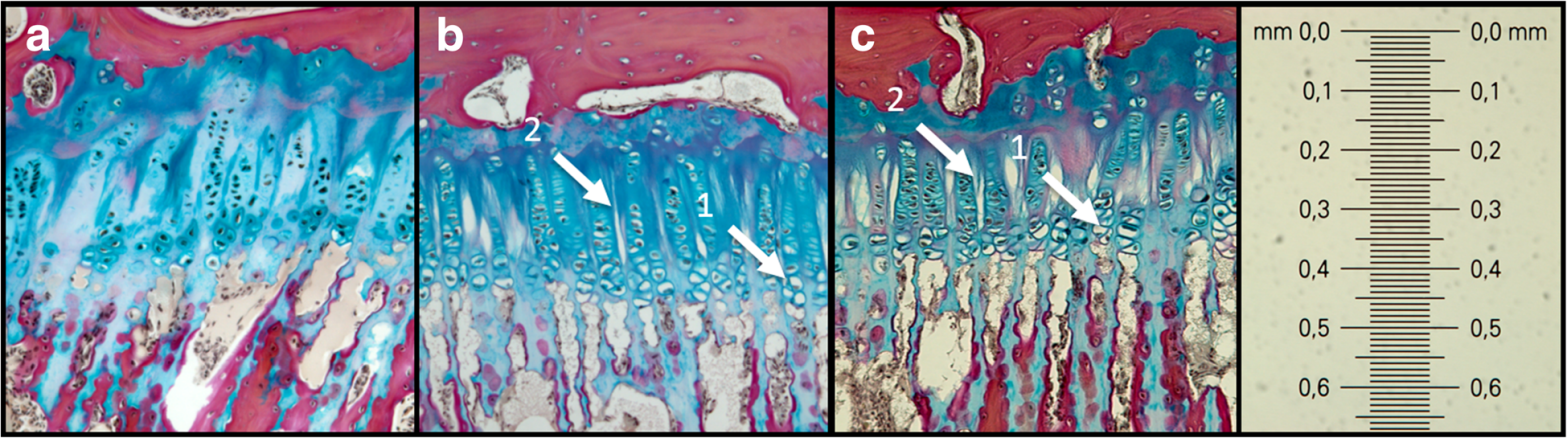
Visualization of the histological results. Control (**a**), flexion (**b**) and extension (**c**) FSUs, where the reduction of intracellular content (1) and extracellular matrix (2) are highlighted with *white arrows*. Sizebar in mm. Haematoxylin-eosin and Alcian *blue* solution stain

The histological morphometric results (Table 4) displayed that the area of unstained parts of the epiphyseal growth zone of loaded FSUs were much larger compared to non-loaded controls. The reduction was seen in both the chondrocytes as well as in the extracellular matrix in the endplate and growth zone area in all loaded FSUs. No disc hernias, fractures or structural deformities were visible. The histological results are visualized in Figs. 4 and 5.

**Table 4:** The area (%) of unstained parts of the epiphyseal growth zone

Group	Mean	Standard deviation	Median	Range
Vibrated	29	12	25	16–53
Flexion	38	10	38	22–53
Extension	21	4	20	16–28
Controls	4	2	4	2–6

Statistical analyze was not conducted due to low sample size

## Discussion

The MRI and histology results related in location of the fatigue effect where both signal reduction and intracellular as well as extracellular substance reduction were visible in the endplate and the growth zone. No disc hernias or fractures were visible. There was no clear difference between the extended and flexed FSUs. These results imply that the signal changes seen on MRI therefore also relate to not only fluid changes but also to both changes and reduction in the intracellular content and in the extracellular matrix.

Pig spine is a well-established experimental model for lumbar pathologies. The material did not display any secondary findings or diseases. The study protocol was chosen to resemble normal every day behavior in both magnitude, frequency and duration as stated in the method section. No signs of failure were detected but potential first signs of fatigue were visible among the loaded FSUs. The load magnitude is probably the most important factor to generate FSU fatigue and failure, considering that earlier studies have showed no fatigue effect even with cycle duration up to 85,000 cycles (Callaghan & McGill 2001).

A majority (94%) of all FSUs have reduced signal in the growth zone and majority (88%) also have increased signal in the vertebral bodies. Among the flexed FSUs a possible trend is that the inferior vertebral body is not affected by the loading as it is among the extended FSUs. The high frequency of growth zone signal reduction is in concurrence with earlier studies that have displayed that the growth zone is the weakest part of porcine FSUs (Baranto et al. 2005a; Baranto et al. 2005b; Thoreson et al. 2010) and the signal reduction is potentially an indication of increased risk of injury as further stated below.

Disc signal reduction is seen in 88% of all discs but a notable result is that both the inferior and superior endplates are affected in the flexion group but among the extended FSUs the superior endplate display no signal reduction. The difference between the flexed and extended FSUs could be due to that the facet joints are more loaded in extension and thereby alter the load affecting the discs and vertebral bodies compared to flexion loading.

Disc degeneration (according to Pfirrman et al. (Pfirrmann et al. 2001)) was only detectable in three FSUs due to that the subjects are young healthy pigs but disc height reduction was seen among 69% in the loaded group. The disc height reduction concurs with earlier results that have displayed a successive disc height reduction from repetitive loading in both experimental loading models and in clinical studies (Gooyers et al. 2012; Dimitriadis et al. 2011; Cheung et al. 2003; Masuoka et al. 2007). In general the loaded FSUs displayed smaller nucleus with less distinct boundaries compared to the controls. The nucleus location of the flexed FSUs were also located more dorsally and among the extended FSUs a more anterior location was situated. No signs of disc hernia were visible in this study in neither the flexed nor the extended group in contrast to earlier findings of a suggested correlation of flexion and/or extension and disc hernias (Wade et al. 2014; Callaghan & McGill 2001) but the dislocation of the nucleus could be a first step towards disc injury.

When comparing the results with an earlier study by Baranto et al. (Baranto et al. 2005a) where the FSUs were loaded in flexion and extension to failure in comparison to the repetitive loading in the present study the results are located in the same area (growth zone and endplates) but differ in severity, and in signal reduction compared to fractures. When comparing the results to an equally matched study by Thoreson et al. (Thoreson et al. 2010) where the porcine FSUs hade underwent 20,000 axial cyclic compressions followed by compression to failure the results also concur with signal reduction in the growth zone and end plates in both study groups. The axial compression group also displayed fractures in these areas secondary to the ultimate loading. This support the theory that the signal differentiation can be seen as a first step towards the injuries that occur in the growth zone and in the end plates. These injuries are often seen among adolescent athletes as ring apophyseal injures and could be due to the increased exposure to both peak and repetitive loads that athletes are subjected to, causing both traumatic and overuse injuries.

The results of the histological examinations were concurrent with the MRI results. No failure injuries such as disc hernia or fractures were seen in the histological examinations due to low load magnitude and load duration. The histological results displayed (Table 4) that the area per cent of unstained parts of the epiphyseal growth zone is largest in flexion specimens and, slightly less, in extension specimens. In the sham-exposed controls only minimal parts of the investigated areas were unstained. The loss of staining is considered to be due to the fatigue due to the exerted mechanical load, resulting in physical and chemical disturbances of the cartilage. The reduction occurred in both the intracellular chondrocyte content as well as in the extracellular matrix in the endplate and in the growth zone of all loaded FSUs (Fig. 5). The reduction could be due to the potential reduction of polysaccharides and hyaluronic acids inside the cell and in the matrix due to mechanical vibration. These are key players in maintaining the cellular and matrix fluid levels and if these are damaged and reduced the fluid levels decrease in the affected tissues.

### Strengths and limitations

A systematic review of the spinal morphological properties, showed that no specific animal is ideal, but young thoracic and lumbar porcine spinal models are considered a suitable animal model for experimental studies (Sheng et al. 2010) and is also a commonly used model (Lotz 2004; Alini et al. 2008). The porcine lumbar vertebra has in contrast to the human vertebra bony endplates, more growth zones, and an epiphyseal plate covering the growth plate instead of the apophyseal ring. However, similar fracture patterns have though been reported between human and porcine spines (Tsai et al. 1997).

An in-vitro experimental model inherently lack of several important factors for maintaining integrity in a high water content structure such as the intervertebral disc. Most notably is the absence of tissue pressure that to some extent counter balance load on the disc. This pressure originated mainly from the blood pressure and respiration. However this is not believed to alter the general outcome, merely offsetting some factors such as viscoelastic behavior. Considering the drawbacks an alternative in vivo model is not plausible due to ethical reasons.

The sectioning process was done in a frozen state with a bench saw to minimize the risk of causing traumatic injuries in the FSUs. However, according to earlier studies and the experience by the same research group only the surface of the slices can be damaged and to reduce this potential effect the histological slices were taken from middle of the sawn slices.

The examination of the MRI was conducted by two of the authors that have long experience of analyzing MRI images in a blinded manner with good inter- and intra-rater results but no ICC validation was conducted. The histology examination was evaluated by an experienced histology specialist but no reliability test was conducted and no statistical analyses were conducted due to the low sample size where the study is considered as a feasibility study.

### Clinical relevance

The results of the study indicate that MRI signal differentiation reflect concurrent histological changes and can be seen as a first step towards the failure injuries that occur in the growth zone and in the endplates in young porcine FSUs. Similar MRI findings as disc signal reduction can be seen clinically on MRI after a 1 h run (Dimitriadis et al. 2011) and spinal injuries in the growth zone and epiphyseal plate are often seen among adolescent athletes (Baranto et al. 2006; Hellstrom et al. 1990; Sward et al. 1990a; Sward et al. 1990b; Granhed & Morelli 1988). The results support that recovery and hysteresis loop are important steps to avoid fatigue and failure injuries.

## Conclusion

Repetitive loading of young porcine FSUs in both extension and flexion causes concurrent MRI and histological changes in the growth zones and endplates which could be a first sign of fatigue and an explanation for disc, apophyseal and growth zone injuries seen among adolescent athletes.

## Abbreviations

EP: Epiphyseal plate
FSU: Functional spinal unit
ICC: Intraclass correlation coefficient
LBP: Low back pain
MRI: Magnetic resonance Imaging
